# Balances: a New Perspective for Microbiome Analysis

**DOI:** 10.1128/mSystems.00053-18

**Published:** 2018-07-17

**Authors:** J. Rivera-Pinto, J. J. Egozcue, V. Pawlowsky-Glahn, R. Paredes, M. Noguera-Julian, M. L. Calle

**Affiliations:** a irsiCaixa AIDS Research Institute, Badalona, Spain; b Universitat de Vic—Universitat Central de Catalunya, Vic, Spain; c Universitat Politècnica de Catalunya, Barcelona, Spain; d Universitat de Girona, Girona, Spain; e Universitat Autónoma de Barcelona, Barcelona, Spain; f Infectious Diseases Service, Hospital Germans Trias i Pujol, Badalona, Spain; University of Colorado Denver

**Keywords:** balances, compositional data, microbiome

## Abstract

High-throughput sequencing technologies have revolutionized microbiome research by allowing the relative quantification of microbiome composition and function in different environments. In this work we focus on the identification of microbial signatures, groups of microbial taxa that are predictive of a phenotype of interest. We do this by acknowledging the compositional nature of the microbiome and the fact that it carries relative information. Thus, instead of defining a microbial signature as a linear combination in real space corresponding to the abundances of a group of taxa, we consider microbial signatures given by the geometric means of data from two groups of taxa whose relative abundances, or balance, are associated with the response variable of interest. In this work we present *selbal*, a greedy stepwise algorithm for selection of balances or microbial signatures that preserves the principles of compositional data analysis. We illustrate the algorithm with 16S rRNA abundance data from a Crohn’s microbiome study and an HIV microbiome study.

**IMPORTANCE** We propose a new algorithm for the identification of microbial signatures. These microbial signatures can be used for diagnosis, prognosis, or prediction of therapeutic response based on an individual’s specific microbiota.

## Abstract (precis)

We propose a new algorithm for the identification of microbial signatures. These microbial signatures can be used for diagnosis, prognosis, or prediction of therapeutic response based on an individual’s specific microbiota.

## INTRODUCTION

Human microbiome research, focused on the study of the microorganisms that live throughout the human body and their role in health and disease, has experienced significant growth in the last few years. High-throughput sequencing technologies have revolutionized this field by allowing the quantification of microbiome composition and function in different environments. Large-scale projects, such as the Human Microbiome Project (1, 2) or MetaHIT (metagenomics of the human intestinal tract), have established standardized protocols for creating, processing, and interpreting metagenomics data (3). However, analysis of microbiome data is still challenging due to, among other reasons, their inherently compositional nature.

High-throughput DNA sequencing generates thousands of sequence reads that, after bioinformatics preprocessing and quality control, are annotated to different microbial species or taxa. An abundance table of counts summarizes the number of sequences per sample of each taxon. The total number of counts per sample, also known as sequencing depth or library size, is highly variable and constrained by the maximum number of sequence reads of the instrument. This total count constraint induces strong dependencies among the abundances of the different taxa; an increase in the abundance of one taxon requires the decrease of the observed number of counts for some of the other taxa so that the total number of counts does not exceed the specified sequencing depth. Moreover, observed raw abundances and the total number of reads per sample are noninformative since they represent only a fraction or random sample of the original DNA content in the environment. These characteristics of microbiome abundance data clearly fall into the notion of compositional data. Compositional data are defined as a vector of strictly positive real numbers with an unknown or uninformative total. Compositional data carry relative information, i.e., information contained in the ratios between components, and the numerical value of each component by itself is irrelevant (4). Except for the fact that microbiome abundance tables contain many zeros, microbiome data fit the definition of compositional data and, as already acknowledged by many authors (5, 6), their analysis requires the use of a proper mathematical theory (7).

Most of the methods proposed for microbiome analysis are intended to address two main issues: first, whether there is a global association between the microbiome and a phenotype of interest; second, which specific taxa are associated with the outcome. Multivariate methods such as PERMANOVA (8, 9), implemented in the *Adonis()* function of the R package *vegan* (10), and MiRKAT (11) address the first issue. The second issue is approached with univariate methods where each taxon is tested for association with the outcome. When the response variable is dichotomous, the testing method is known as differential abundance testing and methods specifically developed for transcriptome sequencing (RNA-Seq) data, such as *DESeq2* (12) and *edgeR* (13), are commonly used. Other methods, such as *ANCOM* (14) and *ALDEx2* (15), have been proposed that acknowledge the compositionality of microbiome data. See a previous report by Weiss et al. for a comprehensive comparison of methods for microbiome differential abundance testing (16).

In this paper we focus on a different issue. The goal of the proposed methodology is to identify microbial signatures, that is, groups of microbial taxa that are predictive of a phenotype of interest. These microbial signatures can be used for diagnosis, prognosis, or prediction of therapeutic response based on an individual’s specific microbiota (17). The identification of microbial signatures involves both modeling and variable selection: modeling the response variable and identifying the smallest number of taxa with the highest prediction or classification accuracy. We present *selbal*, a model selection procedure that searches for a sparse model that adequately explains the response variable of interest. Similarly to forward stepwise linear regression, *selbal* performs multiple regressions a number of times, each time adding a new taxon to the model. Unlike linear regression, the raw variables in *selbal* are not included in a linear equation in real space but are added as part of what is called a “balance” in the compositional data analysis literature, i.e., as part of a particular type of log-contrast.

Mathematically, a compositional balance is defined as follows. Let *X* = (*X*_1_,* X*_2_,* *…,* X*_*k*_) be a composition with *k* components or parts. Given two disjoint subsets of components in* X*, denoted by *X*_+_ and* X*_−_, indexed by *I*_+_ and* I*_−_, and composed of *k*_+_ and *k*_−_ parts, respectively, the balance between *X*_+_ and *X*_−_ is defined as the normalized log ratio of the geometric mean of the values for the two groups of components as follows:

$$B(X_{+},X_{-})=\sqrt{\frac{k_{+}\cdot k_{-}}{k_{+}+k_{-}}}\log\frac{{\left(\Pi_{i\in I_{+}}X_{i}\right)}^{1/k_{+}}}{{\left(\Pi_{j\in I_{-}}X_{j}\right)}^{1/k_{-}}}$$

Expanding the logarithm, we obtain a more usual expression of a balance that is proportional to the difference between the means of the log-transformed variables of the two groups of components as follows:

$$B(X_{+},X_{-})\;\propto \;\frac{1}{k_{+}}\underset{i\in I_{+}}{\sum }\log\;X_{i}-\frac{1}{k_{-}}\underset{j\in I_{-}}{\sum }\log\;X_{j}$$

A compositional balance is a special kind of a log-contrast, defined as a linear combination of the log-transformed components of a composition with the restriction that the coefficients of the linear function add up to zero (4). The importance of working with log-contrast functions or, in particular, with balances, in analyzing compositional data is that this kind of function preserves scale invariance, one of the principles that should be fulfilled in compositional data analysis (4, 7).

Our algorithm for balance selection, *selbal*, starts with a first thorough search of the two taxa whose balance, or log ratio, is most closely associated with the response. Once the first two-taxon balance is selected, the algorithm performs a forward selection process where, at each step, a new taxon is added to the existing balance such that the specified criterion is improved (area under the receiver operating characteristic [ROC] curve [AUC] or mean squared error [MSE]). The algorithm stops when there is no additional variable that improves the current optimization parameter or when the maximum number of components to be included in the balance is achieved. This number is established with a cross-validation (CV) procedure, which is also used to explore the robustness of the identified balance. A more detailed description of the algorithm is given in Materials and Methods.

*selbal* is different from other modeling approaches for microbiome analysis such as MiRKAT (11), which performs an overall association test comparing the microbiome and the phenotype but does not perform model selection.

Model selection for microbial signature identification can also be performed in two separate steps: first, variable selection; second, model building with the selected variables. When the outcome variable is dichotomous, variable selection can be obtained with methods for microbiome differential abundance testing mentioned before, such as *DESeq2* (12), *edgeR* (13), or, in the context of compositional data analysis, *ANCOM* (14) or *ALDEx2* (15). However, it is not clear how to combine the selected variables to obtain the best joint sparse model. This is specially challenging for microbiome analysis, where the compositional nature of microbiome data induces spurious correlations among the variables. We think that a joint procedure that involves both modeling and variable selection, as performed in *selbal*, is more appropriate in this context.

Other authors (18–20) have previously proposed the use of balances for microbiome analysis regarding the construction of an isometric log ratio (ILR) transformation (21), which allows compositional data to be represented in a real Euclidean space, where standard statistical methods can be applied. Silverman et al. (18) and Washburne et al. (19) proposed methods that use microbial phylogenetic information to guide the sequential binary partition in the construction of a particular ILR transformation. This phylogenetically driven ILR transformation would help to detect relevant evolutionary factors or phylogenetically related bacterial groups (clades) related to host-microbiome interactions (18, 19). In the method proposed by Morton et al. (20), instead of using phylogenetic information, they use the response variable to define the binary sequential partitions of the ILR transformation. *selbal* is different from these methods in the following ways: first, only one balance is considered and not a sequence of balances in *selbal*; second, the purpose of the selected balance is classification or prediction and not a new representation of the data.

As with any other compositional data method, one important issue that *selbal* addresses before the searching algorithm can be applied is that of how to deal with the large amount of zeros that are typically present in microbiome data sets. Their treatment is different depending on whether they represent essential or rounded zeros (22). In microbiome analysis, an essential or structural zero represents the absolute absence of a taxon in a sample, e.g., because the microbe is unable to live in that environment. In dealing with essential zeros, samples are considered to belong to two distinct subpopulations according to the presence or absence of a zero. On the other hand, rounded zeros arise because of insufficient sampling depth: they correspond to taxa in such a small proportion in the sample that they were not picked during the sequencing process. The common practice for the treatment of rounded zeros is their replacement by a small positive value. This replacement can be implemented in different ways, including replacing the zeros by a constant, adding a constant to all values in the data set, and using more-sophisticated methods for zero replacement that are designed to preserve as much as possible the covariance structure of the initial data. Though the user can perform other zero replacements before using *selbal* (18, 20), the default option in *selbal* is geometric Bayesian multiplicative replacement (GBM) (23) (described in more detail in Materials and Methods).

Another important issue in microbiome analysis is sampling variance. As discussed by Gloor et al. (24), microbiome data, as with any other kind of high-throughput sequencing data, are subject to high levels of variability or uncertainty that should be conveniently treated. The number of reads obtained in an experiment represents just an instance from a random sample of the true microbial composition in the environment of interest. The same protocol applied twice to the same sample would provide different microbial compositions. This imprecision, which arises during the library preparation and sequencing process, is larger for taxa of low abundance and should be taken into account when dealing with observed zeros. One way to handle this variability is by modeling the read counts per sample as multinomial or negative binomial, which implies that the vectors of relative abundances of the different taxa follow approximately a Dirichlet distribution. Then, using a Dirichlet Monte Carlo sampling approach, we can obtain multiple instances of the posterior distribution of the relative abundances determined for each sample (25). By multiplying the relative abundances of each sample by the observed total number of counts, we obtain multiple abundance tables which can be analyzed with *selbal*. The comparison of the microbial signatures obtained from these new Monte Carlo abundance tables to the microbial signature obtained with the initial table can be used to evaluate the robustness of the initial result.

The remainder of this paper is organized as follows. In the next section, we illustrate the proposed algorithm with a Crohn’s disease (CD) microbiome study and an HIV microbiome study. Some discussions and suggestions for future work are provided in the Discussion. In Materials and Methods, we present the detailed description of the algorithm. *selbal* is accessible as an R package in GitHub (https://github.com/UVic-omics/selbal), where the data sets and scripts to reproduce this work are also available.

## RESULTS

We illustrate the proposed methodology for use with microbiome compositions at the genus level for a Crohn’s disease study (26) and an HIV microbiome study (27). We did not perform the bioinformatics processing of the sequences (with Mothur or Qiime) but took the processed operational taxonomic unit (OTU) tables available in Qiita and Bioproject repositories (accession numbers provided below). We performed additional filtering and agglomeration steps to obtain the abundance tables at the genus level. The scripts to obtain those genus-level abundance tables are available at GitHub.

### Microbiome and Crohn’s disease.

Crohn’s disease (CD) is an inflammatory bowel disease that has been linked to microbial alterations in the gut (26, 28). We use data from a large pediatric CD cohort study (26) to illustrate the proposed methodology for identification of microbial signatures. Microbiome data from 16S rRNA gene sequencing and QIIME 1.7.0 bioinformatics processing were downloaded from Qiita https://qiita.ucsd.edu (study identifier [ID]: 1939). Only patients with Crohn’s disease (*n* = 662) and those without any symptoms (*n* = 313) were analyzed. The OTU table was agglomerated to the genus level, resulting in a matrix with 48 genera and 975 samples.

The goal of *selbal* analysis is to identify a microbial signature for Crohn’s disease that is able to discriminate between CD and non-CD individuals. This microbial signature is defined by two groups of taxa whose relative abundances, or balance, are associated with Crohn’s disease status.

**FIG 1:**
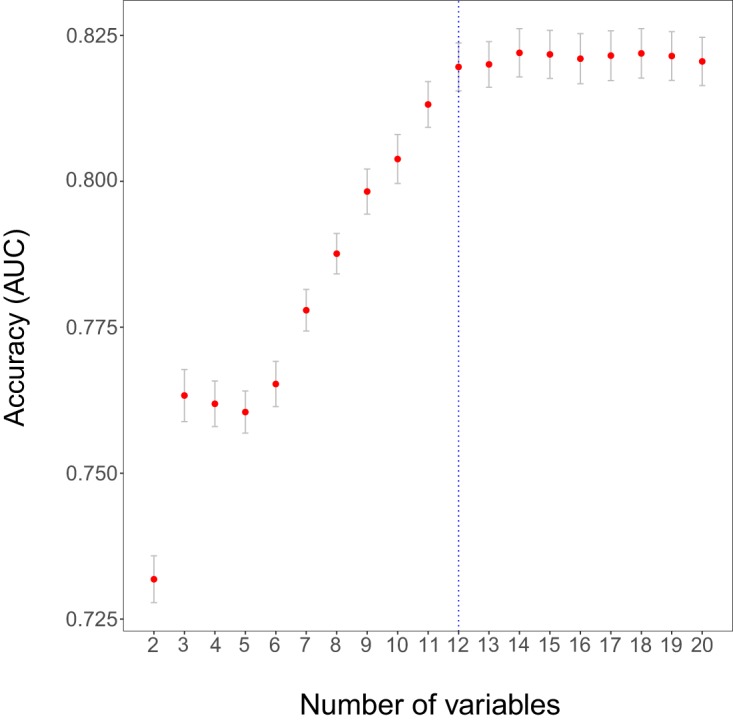
Mean area under the ROC curve (AUC) as a function of the number of components included in the balance in the cross-validation process for Crohn’s disease. The optimal number of components according to the “1se rule” is highlighted with a vertical dashed line.

As explained in Materials and Methods, we first ran a cross-validation (CV) process with the function selbal.cv*()* that helped us to determine the optimal number of taxa to be included in the balance. Figure 1 provides the mean AUC and standard error of the balances obtained in the CV process as a function of the number of taxa. In this case, and following the 1se rule, the optimal number of taxa is 12.

Once the number of taxa is determined, we apply the main function *selbal()* to the whole data set, with the number of taxa *C* = 12, and obtain what we call the "global balance."

**FIG 2:**
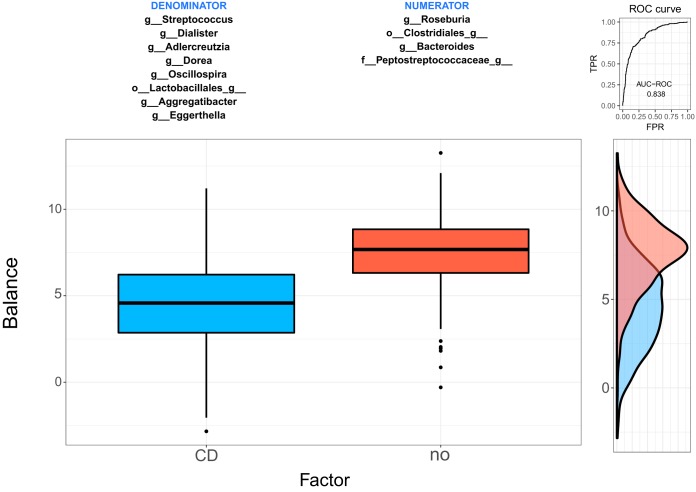
Description of the global balance for Crohn’s disease. The two groups of taxa that form the global balance are specified at the top of the plot. The box plot represents the distribution of the balance scores for CD and non-CD individuals. The right part of the figure contains the ROC curve with its AUC value (0.838) and the density curve for each group.

The two groups of taxa defining the global balance, or microbial signature, for Crohn’s disease are *X*_+_ = {*g_Roseburia*, *o_Clostridiales_g_*, *g_Bacteroides*, *f_Peptostreptococcaceae_g_*} and *X*_−_ = {*g_Dialister*, *g_Dorea*, *o_Lactobacillales_g_*, *g_Eggerthella*, *g_Aggregatibacter*, *g_Adlercreutzia*, *g_Streptococcus*, *g_Oscillospira*}. Figure 2 presents the distribution of the microbial signature values for CD and non-CD individuals. Patients with Crohn’s disease have lower balance scores than controls, which means that there are lower relative abundances of taxa in group *X*_+_ than in group* X*_−_. *Bacteroides* and *Clostridiales* have also been previously identified as less abundant in Crohn’s disease individuals than in controls (26). The discrimination value of the identified balance is important, with an apparent AUC value of 0.838. However, this apparent AUC is known to overestimate the discrimination value of the microbial signature, since it has been measured on the same data set that was used to build the model. A more accurate estimation is obtained from the CV process, which provides a cv-AUC of 0.819, a very good discrimination value.

### Robustness of the selected global balance.

**FIG 3:**
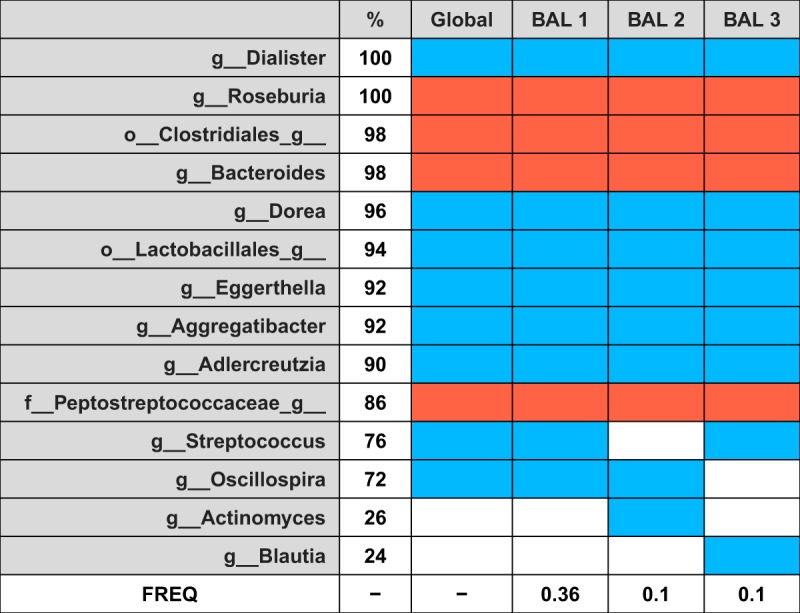
Cross-validation (CV) results for Crohn’s disease study: most frequent taxa and most frequent balances selected in the CV procedure compared to the global balance obtained with the whole data set. Colored rectangles indicate if the component is in the numerator of the balance (BAL) (red), in the denominator (blue), or not included (white). FREQ, frequency.

CV can also help us to assess the robustness of the proposed global balance. In Fig. 3, we summarize the different balances obtained with 12 taxa in the CV process. On the one hand, we have the frequency of the different CV balances and, on the other, the frequency of selection of each taxon. Rows represent the most frequent taxa, with their percentage of selection given in the second column; the third column represents the global balance, that is, the balance obtained using all the samples; and the last three columns represent the three most frequent balances selected in the CV procedure. Colored rectangles indicate whether the taxon is in the numerator of the balance (red) or in the denominator (blue) or not included (white). The last row indicates the proportion of times each balance has been selected as optimal in the CV procedure. From the data presented in Fig. 3, it follows that the identified global balance for Crohn’s disease is very robust; the global balance coincides with the balance most frequently found in the CV process, which turned out to be the optimal balance 36% of the time. Moreover, the taxa which form the global balance are also those most frequently selected in the CV procedure.

An alternative approach for exploring the robustness of the selected global balance is by implementing Monte Carlo sampling from a Dirichlet distribution prior to microbiome signature identification with *selbal*. This process returns a set of different microbiome signatures that can be compared with the global balance. We performed this strategy with Crohn’s microbiome data with 100 sampling iterations and obtained balances highly concordant with the proposed global balance (see Fig. S1 in the supplemental material). Similar results were obtained with two zero replacement strategies, a constant of 1 added to all values, and geometric Bayesian multiplicative replacement (GBM) (23).

10.1128/mSystems.00053-18.1 FIG S1  Results of a Monte Carlo sampling implementation from a Dirichlet distribution prior performed in order to evaluate Crohn’s disease results, taking the variability and uncertainty of raw reads into account. The table summarizes the balances obtained after 100 sampling iterations. Download FIG S1, PDF file, 0.1 MB. Copyright © 2018 Rivera-Pinto et al. 2018 Rivera-Pinto et al. This content is distributed under the terms of the Creative Commons Attribution 4.0 International license.

### Comparison with other approaches.

Using the Crohn’s disease data set, we compared the classification accuracy of *selbal* with that of strategies employing two steps: first, variable selection; second, model building. For the variable-selection step, we considered *DESeq2*, *edgeR*, *ANCOM*, and *ALDEx2*, and then we built a model or microbial signature with the selected variables. The model is a linear combination of the selected variables for *DESeq2*, *edgeR*, and *ANCOM*, whereas for *ALDEx2* the model is defined as a linear combination of the selected variables, previously transformed according to the centered log-ratio transformation (15). *selbal* cannot be compared with these methods in terms of false-discovery rate (FDR) and power because the goal of *selbal* is not to identify all taxa that are associated with the response but to obtain the best sparse model to predict the response. In a cross-validation process, we measured the test prediction accuracy and sparsity of the models (microbial signatures) obtained with each method. The results are given in Table 1, and in Fig. S2 we can see the variability of cv-AUC for the different methods.

10.1128/mSystems.00053-18.2 FIG S2  Comparison of the discrimination accuracies of different methods for Crohn’s disease status. Each box plot summarizes the AUC values obtained in a 5-fold procedure repeated 10 times. Download FIG S2, PDF file, 0.04 MB. Copyright © 2018 Rivera-Pinto et al. 2018 Rivera-Pinto et al. This content is distributed under the terms of the Creative Commons Attribution 4.0 International license.

**TABLE 1:** Comparison of model complexity and discrimination accuracy of microbial signatures for Crohn’s disease status^a^

Method	Median no. of taxa	Mean cv-AUC
*selbal*	12	0.8196
DESeq2	33	0.7752
edgeR	34	0.7721
ANCOM	5	0.7125
ALDEx2	31	0.8156

a For each method, the table indicates the median number of taxa included in the model and the mean cv-AUC for 10 iterations of a 5-fold cross-validation process.

*selbal* and *ALDEx2* are the methods with the best classification accuracy, but *selbal* is more parsimonious, which is also a desirable feature of microbial signatures. *selbal* obtains discrimination accuracy with only 12 taxa that is similar to that obtained with *ALDEx2* with 31 taxa. *DESeq2* and *edgeR* provide similar results: high numbers of selected taxa but lower classification accuracy. This suggests that among the variables selected by *DESeq2* and *edgeR*, there are some false positives. *ANCOM* is the best in terms of parsimony; it selects the smallest number of variables, with classification accuracy comparable to that of *DESeq2* and *edgeR*. This is in accordance with previous simulation studies (16) that indicated that *ANCOM* has very low FDR and comparable power to other methods. These results cannot be generalized since they reflect the behavior of the methods with only one specific data set. A more general conclusion would require a comprehensive simulation study.

### Microbiome and HIV infection.

Understanding the role of the gut microbiome in HIV-1 infection may help to design novel interventions to improve HIV-1-associated immune dysfunction. We considered a cross-sectional HIV microbiome study conducted in Barcelona, Spain, that included both HIV-infected subjects and HIV-negative controls (27). Microbiome data were obtained from a MiSeq 16S rRNA sequence and bioinformatically processed with Mothur (29) and are available at BioProject (PRJNA307231). After applying abundance filters and agglomerating taxa to the genus level, microbiome abundance data are summarized in a matrix of raw abundances for 155 samples and 60 different genera.

The main goal of this analysis is to find a microbial signature for HIV status, that is, two groups of taxa whose relative abundance or balance data are able to discriminate between HIV-positive and HIV-negative individuals. As reported by Noguera-Julian et al. (27), a possible confounder in HIV microbiome studies is the HIV risk factor MSM (men who have sex with men versus non-MSM). *selbal* algorithm implements a regression model which allows adjustment for other variables. Thus, we applied the algorithm to *Y* = HIV status and *X* = microbiome abundance at the genus level, adjusted by *Z* = MSM factor.

According to the cross-validation (CV) procedure implemented with function selbal.cv*()*, the optimal number of components to be included in the balance is 2 (Fig. S3). The microbiome signature for HIV status identified with *selbal* is given by the log ratio between the abundance of a taxon of the family *Erysipelotrichaceae* and of unknown genus and a taxon of the family *Ruminococcaceae* and of unknown genus (Fig. S4). HIV-negative individuals are associated with lower balance values, most of them negative, that is, with larger relative abundances of *Ruminococcaceae* than of *Erysipelotrichaceae*, while HIV-positive individuals have heterogeneous balance values. The discrimination accuracy of this balance is moderate, with an AUC of 0.786 on the whole sample and a mean cross-validation AUC of 0.674 measured on the test data sets given MSM status. Figure S5 shows the result of the cross-validation procedure. The balance identified with the whole data set is that most frequently identified in the cross-validation procedure, appearing 44% of the time, an indicator of robustness for the proposed global balance.

10.1128/mSystems.00053-18.3 FIG S3  Mean area under the ROC curve (AUC) as a function of the number of components included in the balance in the iterative cross-validation process for HIV-positive (HIV^+^) and HIV^−^ individuals, taking the MSM factor as a possible confounder. The optimal number of components according to the “1se rule” is highlighted with a vertical dashed line. Download FIG S3, PDF file, 0.03 MB. Copyright © 2018 Rivera-Pinto et al. 2018 Rivera-Pinto et al. This content is distributed under the terms of the Creative Commons Attribution 4.0 International license.

10.1128/mSystems.00053-18.4 FIG S4  Description of the global balance for HIV study. The two groups of taxa that form the global balance are specified at the top of the plot. The box plot represents the distribution of the balance scores for noninfected (Neg) and infected (Pos) individuals. The right part of the figure contains the ROC curve with its AUC (0.786) value and the density curve for each group. Download FIG S4, PDF file, 0.1 MB. Copyright © 2018 Rivera-Pinto et al. 2018 Rivera-Pinto et al. This content is distributed under the terms of the Creative Commons Attribution 4.0 International license.

10.1128/mSystems.00053-18.5 FIG S5  Cross-validation (CV) results for HIV study: most frequent taxa and most frequent balances selected in the CV procedure compared to the global balance obtained with the whole data set. Colored rectangles indicate if the component is in the numerator of the balance (red) or is in the denominator (blue) or is not included (white). Download FIG S5, PDF file, 0.1 MB. Copyright © 2018 Rivera-Pinto et al. 2018 Rivera-Pinto et al. This content is distributed under the terms of the Creative Commons Attribution 4.0 International license.

### Microbiome and soluble CD14 inflammation marker.

Acute inflammation and chronic inflammation typically occur after HIV infection. Even patients administered antiretroviral medications and with an undetectable viral load present with chronic inflammation, which may cause tissue damage and is associated with many chronic diseases (30). In this context, there is great interest in defining possible interventions involving modifications of the gut bacterial environment which may reduce inflammation in HIV patients (31, 32). This requires a good understanding of the association between gut microbial composition and several inflammation markers. In this case, we focus on immune markers related to chronic inflammation: the levels of soluble CD14 (sCD14), which was measured for a subset of samples (*n* = 151). We apply *selbal* to search for a microbial signature that is predictive of an sCD14 inflammation marker. According to the cv-MSE (Fig. S6), the optimal number of components to be included in the model is four. The balance that is identified as that most closely associated with sCD14 is composed of two taxa in the numerator, *X*_+_ = {*g_Subdoligranulum*, *f_Lachnospiraceae_g_Incertae_Sedis*}, and two in the denominator, *X*_−_ = {*f_Lachnospiraceae_g_unclassified*, *g_Collinsella*}. The association is moderate, with *R* = 0.53. Since sCD14 data are continuous, we represent the result with a scatter plot of the balance values and sCD14 values. We observe that higher balance scores are associated with higher sCD14 values (Fig. S7). The robustness of the selected balance can be evaluated through the results of the CV procedure (Fig. S8). We see that the proposed global balance is also the one that has been the most frequently (34% of the time) selected in the CV. The four taxa defining the global balance correspond to the top 4 most frequently selected in the cross-validation. These results emphasize the robustness of the selected global balance.

10.1128/mSystems.00053-18.6 FIG S6  Mean squared error (MSE) as a function of the number of components included in the balance in the cross-validation process for sCD14 analysis. The optimal number of components according to the “1se rule” is highlighted with a vertical dashed line. Download FIG S6, PDF file, 0.03 MB. Copyright © 2018 Rivera-Pinto et al. 2018 Rivera-Pinto et al. This content is distributed under the terms of the Creative Commons Attribution 4.0 International license.

10.1128/mSystems.00053-18.7 FIG S7  Description of the global balance for the sCD14 study. The two groups of taxa that form the global balance are specified at the top of the plot. Below, the regression model is represented with the sCD14 values defined in the *y* axis and the balance value in the *x* axis. Higher balance scores are associated with higher levels of sCD14. Download FIG S7, PDF file, 0.1 MB. Copyright © 2018 Rivera-Pinto et al. 2018 Rivera-Pinto et al. This content is distributed under the terms of the Creative Commons Attribution 4.0 International license.

10.1128/mSystems.00053-18.8 FIG S8  Cross-validation (CV) results for sCD14 study: most frequent taxa and most frequent balances selected in the CV procedure compared to the global balance obtained with the whole data set. Colored rectangles indicate if the component is in the numerator of the balance (red) or in the denominator (blue) or not included (white). Download FIG S8, PDF file, 0.1 MB. Copyright © 2018 Rivera-Pinto et al. 2018 Rivera-Pinto et al. This content is distributed under the terms of the Creative Commons Attribution 4.0 International license.

## DISCUSSION

The identification of microbial signatures that are predictive of a variable of interest is an essential step toward the translation of microbiome research to clinical practice. In this work, we present *selbal*, a greedy stepwise algorithm for the identification of microbial signatures consisting of two groups of taxa whose relative abundances, or balance, are predictive of the outcome. Working with balances and, in general, with log-contrast functions preserves the scale-invariant principle for compositional data analysis.

In the Crohn’s disease study considered in this work, *selbal* outperformed methods commonly used in microbiome analysis, such as *DESeq2* and *edgeR*, in terms of discrimination accuracy. With respect to methods for compositional data, *selbal* performs much better than ANCOM and similarly to *ALDEx2* in terms of classification accuracy, but *selbal* is more parsimonious.

*selbal* overcomes the problem of differences in sample size that is usually accommodated with different methods based on count normalization, rarefaction, or transformation into proportions. These normalization techniques are controversial since they may have an important impact on the analysis (16, 33, 34). The only way in which data are altered in *selbal* is at the zero imputation stage, which is required because of the use of logarithms and ratios in the definition of balances. This replacement of zeros by positive numbers is performed under the assumption that the observed zeros represent rounded zeros, that is, that all taxa are present in all the samples but some of them are not detected because of low abundance and insufficient sampling depth. However, it is not clear how the imputation method and the presence of structural zeros (absence of the taxa in the sample) may influence the results. Future research will focus on the treatment of zeros, with the aim of more precisely evaluating if zeros are rounded or structural, and on selecting the best replacement method.

The technical variability of microbiome sequencing data should be taken in consideration. The effects of this uncertainty on the results of *selbal* can be explored through Monte Carlo sampling from a Dirichlet distribution (25) prior to microbiome signature identification with *selbal*.

A limitation of *selbal* is that the greedy algorithm does not guarantee that the global optimum will be found. Due to the computational cost, *selbal* does not explore the whole balance space; the method for selecting the optimal balance is suboptimal and may be improved. In this respect, the iterative CV process included in the *selbal* algorithm is useful for exploring the robustness of the result. The degree of concordance between the balances obtained in the CV process and the global balance can provide reasonable evidence to support the optimality of the global balance. Exploring possible alternative approaches in the search of the optimal balance is another topic of future research.

## MATERIALS AND METHODS

Let *X* = (*X*_1_,* X*_2_,* *…,* X*_*k*_) be a composition, that is, a vector of strictly positive real numbers that carry relative information. Given two disjoint subsets of components in* X*, denoted by *X*_+_ and* X*_−_, indexed by *I*_+_ and* I*_−_, and composed of *k*_+_ and *k*_−_ features, respectively, the balance between *X*_+_ and *X*_−_ is defined as the normalized log ratio of the geometric mean of the two groups of components as follows:

$$B(X_{+},X_{-})=\sqrt{\frac{k_{+}\cdot k_{-}}{k_{+}+k_{-}}}\;\log\;\frac{{\left(\Pi_{i\in I_{+}}X_{i}\right)}^{1/k_{+}}}{{\left(\Pi_{j\in I_{-}}X_{j}\right)}^{1/k_{-}}}$$

Expanding the logarithm, we obtain the result that a balance is proportional to the difference between the arithmetic means of the log-transformed variables of the two groups of components as follows:

$$B(X_{+},X_{-})\;\propto \;\frac{1}{k_{+}}\underset{i\in I_{+}}{\sum }\log\;X_{i}-\;\frac{1}{k_{-}}\underset{j\in I_{-}}{\sum }\log\;X_{j}$$

The second expression is preferable from a computational point of view and is the one implemented in the proposed algorithm.

Given *Y*, a response variable, which can be either numeric or dichotomous, a composition *X* = (*X*_1_,* X*_2_,* *…,* X*_*k*_), and additional covariates *Z* = (*Z*_1_,* Z*_2_,* *…,* Z*_*r*_), the goal of the algorithm is to determine two subcompositions of *X*, *X*_+_ and *X*_−_, indexed by *I*_+_ ⊂ {1, 2,…,* k*} and *I*_−_ ⊂ {1, 2,…,* k*}, respectively, so that the balance *B*(*X*_+_, *X*_−_) between *X*_+_ and *X*_−_ is highly associated with *Y* after adjustment for covariates *Z*. Depending on the nature of the dependent variable, the association can be defined in several ways.

For a continuous variable* Y*, the optimization criterion is defined as minimization of the MSE of the linear regression model as follows:

$$Y=\beta_{0}+\beta_{1}B(X_{+},X_{-})+\gamma \prime Z$$

For a dichotomous variable* Y*, we fit the logistic regression model as follows:

$$\text{logit}(Y)=\beta_{0}+\beta_{1}B(X_{+},X_{-})+\gamma \prime Z$$

In this case, we consider three possible optimization criteria corresponding to the maximization of the area under the ROC curve (default option), the maximization of the explained variance (35), or the discrimination coefficient (36).

### Main function: *selbal()*.

The main function of the proposed algorithm to detect the most closely associated balance is called *selbal()* and employs the following three steps.

### Step 0: zero replacement.

The initial matrix of counts in a microbiome study, denoted *X̃*, typically contains many zeros that must be treated prior to using *selbal* algorithm. Though the user can perform other zero replacements before using *selbal* (18, 20), the default option in *selbal* is geometric Bayesian multiplicative replacement (GBM) (23) as implemented in the *cmultRepl()* function of the R package *zCompositions* (37). GBM performs Bayesian estimation of the zero values, assuming a Dirichlet model and a multiplicative modification of the nonzero values, so that both the ratios between parts and the total sum of the initial vector before the replacement are preserved. GBM performs better than other Bayesian multiplicative replacements assuming a Dirichlet distribution (23). *selbal* also provides the option of adding a value of 1 to all values in the data set. The resulting matrix with zeros replaced by positive values is denoted by *X*.

### Step 1: optimal balance between two components.

The algorithm evaluates exhaustively all possible balances composed of only two components, that is, all balances of the following form:

$$B_{ij}=\sqrt{\frac{1}{2}}\;\left[\log\left(X_{i}\right)-\text{log}\left(X_{j}\right)\right]\text{ for }i,\;j\;\in \left\{1, . . .,\;k\right\},\;i\neq j\;$$

Each two-component balance (*B_ij_*) is tested for association with the response variable *Y* with one of these regression models as follows:

$$Y=\beta_{0}+\beta_{1}B_{ij}+\gamma \prime Z,\;\text{for a continuous response, or}$$

$$\text{logit}(Y)=\beta_{0}+\beta_{1}B_{ij}+\gamma \prime Z,\text{for a dichotomous variable}\;Y.$$

The balance that maximizes the optimization criteria (MSE or AUC) is selected and denoted by* B*_1_.

Note that in defining a balance for a pair of components (*X*_*i*_, *X*_*j*_), there are two options that differ only in their signs but provide the same association with the response:

$$B_{ij}=\sqrt{\frac{1}{2}}\;\left[\log\left(X_{i}\right)-\text{log}\left(X_{j}\right)\right]\;and\;B_{ji}=\sqrt{\frac{1}{2}}\;\left[\log\left(X_{j}\right)-\text{log}\left(X_{i}\right)\right]$$

*selbal* returns the balance whose regression coefficient is positive.

### Step *s*: optimal balance—adding a new component.

For *s* = >1 and until the stop criterion is fulfilled, let *B*^(*s* − 1)^ be the balance defined in the previous step:

$$\;B^{\left(s-1\right)}\;\propto \;\frac{1}{k_{+}^{\left(s-1\right)}}\underset{i\in I_{+}^{\left(s-1\right)}}{\sum }\text{log}(X_{i})-\;\frac{1}{k_{-}^{\left(s-1\right)}}\underset{j\in I_{-}^{\left(s-1\right)}}{\sum }\text{log}(X_{j})$$

where $I_{+}^{\left(s-1\right)}$ and $I_{-}^{\left(s-1\right)}$ are two disjoint subsets of indices in (1,* *.* *.* *.,* k*), with $k_{+}^{\left(s-1\right)}$ and $k_{-}^{\left(s-1\right)}$ elements, respectively.

For each of the remaining variables, *X*_*p*_, not yet included in the balance, *p* ∉ $[I_{+}^{\left(s-1\right)}\;⋃{\;I}_{-}^{\left(s-1\right)}]$, the algorithm considers the balance that is obtained by adding log(*X_p_*) to the positive part of the previous balance *B*^(*s*−1)^

$$B_{p}^{\left(s+\right)}\propto \left\{\frac{1}{k_{+}^{\left(s-1\right)}+1}\left[\underset{i\in I_{+}^{\left(s-1\right)}}{\sum }\log\left(X_{i}\right)+\log(X_{p})\right]-\;\frac{1}{k_{-}^{\left(s-1\right)}}\underset{j\in I_{-}^{\left(s-1\right)}}{\sum }\text{log}(X_{j})\right\}$$

and the balance that is obtained by adding log(*X_p_*) to the negative part of *B*^(*s*−1)^

$$B_{p}^{\left(s-\right)}\propto \{\frac{1}{k_{+}^{\left(s-1\right)}}\underset{i\in I_{+}^{\left(s-1\right)}}{\sum }\log\left(X_{i}\right)-\;\frac{1}{k_{-}^{\left(s-1\right)}+1}\left[\underset{j\in I_{-}^{\left(s-1\right)}}{\sum }\text{log}(X_{j})+\log(X_{p})]\right\}$$

Each of these pairs of balances, $B_{p}^{\left(s+\right)}$ and$\;B_{p}^{\left(s-\right)}$, for each of the remaining variables, *X*_*p*_, is tested for association with the response variable through one of these two regression models, where *B* denotes the balance tested:

$$Y=\beta_{0}+\beta_{1}B+\gamma \prime Z,\;for\;a\;continuous\;response,\;or$$

$$\text{logit}(Y)=\beta_{0}+\beta_{1}B+\gamma \prime Z,\;for\;a\;dichotomous\;\mathrm{var}iable\;Y.$$

The balance that maximizes the optimization criterion defines the new balance* B*^(*s*)^.

### Stop criteria.

There are two stopping rules: the iterative algorithm stops when value corresponding to the the improvement of the optimization parameter is lower than a specified threshold *th.imp* (default *th.imp* = 0) or when the specified maximum number of components, *C*, has been included in the balance (default *C* = 20).

### Iterative cross-validation: selbal.cv*()*.

An iterative cross-validation procedure is implemented in selbal.cv*()* function with two goals: (i) to identify the optimal number of components to be included in the balance and (ii) to explore the robustness of the global balance identified with the whole data set.

Let *M* be the number of iterations (default* M* = 10), *K* the number of folds in the cross-validation (default* K* = 5), and *C* the maximum number of variables or components included in a balance (default *C* = 20).

At each iteration of* m* ∈ {1,* *…,* M*}, the data are divided into *K* folds, $D_{1}^{m}, . . .,\;D_{K}^{m}$.

For each* k* ∈ {1,* *…,* K*}, *selbal()* is applied to the training data set, $U_{j\neq k}D_{j}^{m}$, and the optimal balance with *C* = 20 variables is obtained, $B_{k}^{m}(20)$. Since *selbal()* is a forward selection process where variables are included sequentially at every step, we have a sequence of balances, including from *C* = 2 to *C* = 20 variables:

$$B_{k}^{m}\left(2\right),\;B_{k}^{m}(3),.\;.\;.,B_{k}^{m}(20)$$

The classification accuracy (MSE or AUC) of these balances is measured on the test data set, $D_{k}^{m}$, giving a sequence of accuracy measures for each number of variables included in the balance:

$$MSE_{k}^{m}\left(2\right),\;MSE_{k}^{m}(3),\;.\;.\;.,MSE_{k}^{m}(20)$$

and similarly for AUC.

### Optimal number of components.

For each number of components c ∈ {2, . . ., C} we have *K* × *M* measures of accuracy, MSE or AUC. The mean and the standard error are computed and are represented in a plot (Fig. 1).

Similarly to the cross-validation process in LASSO for finding the optimal penalization parameter lambda (38), we follow the "1se strategy" and define the optimal number of variables included in the balance (*k_opt_*) as the lowest number whose mean MSE is within 1 standard error of the minimum mean MSE (or whose mean AUC is within 1 standard error of the maximum mean AUC). Usually, the 1se strategy provides sparser models than taking the minimum mean MSE (or maximum mean AUC), with very similar accuracy. This 1se strategy is the default option in *selbal*, but there is also the possibility of determining the optimal number of variables as the value reaching the optimum (minimum mean MSE or maximum mean AUC).

### Global balance.

Once the optimal number of components *k_opt_* has been determined, we apply the main function *selbal()* to the whole dataset, with the number of taxa *C* = *k_opt_*, and obtain what we call the global balance.

### Robustness of the result.

Any method that requires variable selection may result in overfitting. In order to explore the robustness of the global balance and the variables that form it, we retrieve all the balances with *k_opt_* components obtained in the cross-validation process $B_{k}^{m}\left(k_{opt}\right),\;k\;\in \left\{1, . . .,\;K\right\},\;m\;\in \;\{1, . . .,\;M\}$ and compare them with the global balance. We summarize these cross-validation balances in two different ways: per balance and per variable. We provide the relative frequencies of the different balances and the proportion of times that each taxon has been included into a balance. This information, available in the output of *selbal.cv()*, is summarized in a table such as that shown in Fig. 3.

This cross-validation process can also be used to obtain the cross-validation accuracy, defined as the mean MSE or mean AUC of the balances obtained in the CV process that have the same number of variables as the global balance: $\underset{k,m}{\mathrm{mean}}\left[{\text{MSE}}_{k}^{m}(k_{opt})\right]$ or $\underset{k,m}{\mathrm{mean}}\left[{\text{AUC}}_{k}^{m}(k_{opt})\right].$

### Data availability.

*selbal* is accessible as an R package in Github (https://github.com/UVic-omics/selbal), where the data sets and scripts to reproduce this work are also available. Microbiome data were obtained from a MiSeq 16S rRNA sequence and bioinformatically processed with Mothur (29) and are available at BioProject (https://www.ncbi.nlm.nih.gov/bioproject/) (accession number PRJNA307231; SRA accession number SRP068240).
