# Supplementary material for: Balances: a New Perspective for Microbiome Analysis
Source: mSystems. 2018 Jul 17;3(4):e00053-18. doi: 10.1128/mSystems.00053-18 (PMC6050633; doi:10.1128/mSystems.00053-18)
Supplement: FIG S1 [file sys004182245sf1.pdf]

Figure S1

	%	Global	BAL 1	BAL 2	BAL 3
g__Bacteroides	100				
g__Dorea	100				
g__Dialister	100				
o__Lactobacillales_g__	100				
g__Roseburia	100				
g__Streptococcus	100				
o__Clostridiales_g__	95				
f__Peptostreptococcaceae_g__	87				
g__Adlercreutzia	81				
g__Eggerthella	81				
g__Oscillospira	80				
g__Aggregatibacter	58				
f__Ruminococcaceae_g__	37				
g__Actinomyces	29				
g__Veillonella	10				
FREQ	–	–	0.28	0.08	0.08
