# Supplementary figures and images for: Balances: a New Perspective for Microbiome Analysis
Source: mSystems. 2018 Jul 17;3(4):e00053-18. doi: 10.1128/mSystems.00053-18 (PMC6050633; doi:10.1128/mSystems.00053-18)

Figure S2

Method comparison

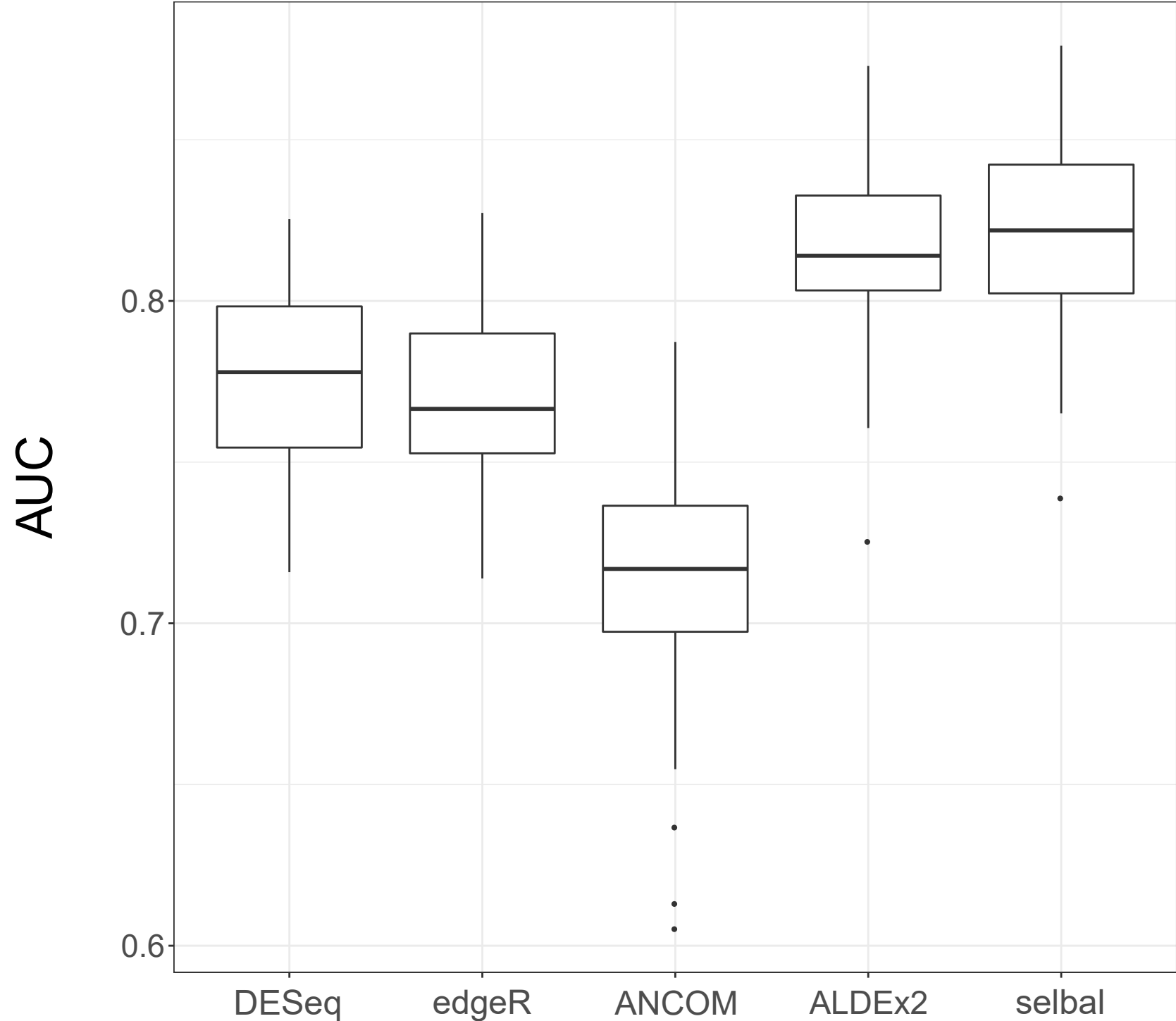

Supplement: FIG S2 [file sys004182245sf2.pdf]

# Figure S3

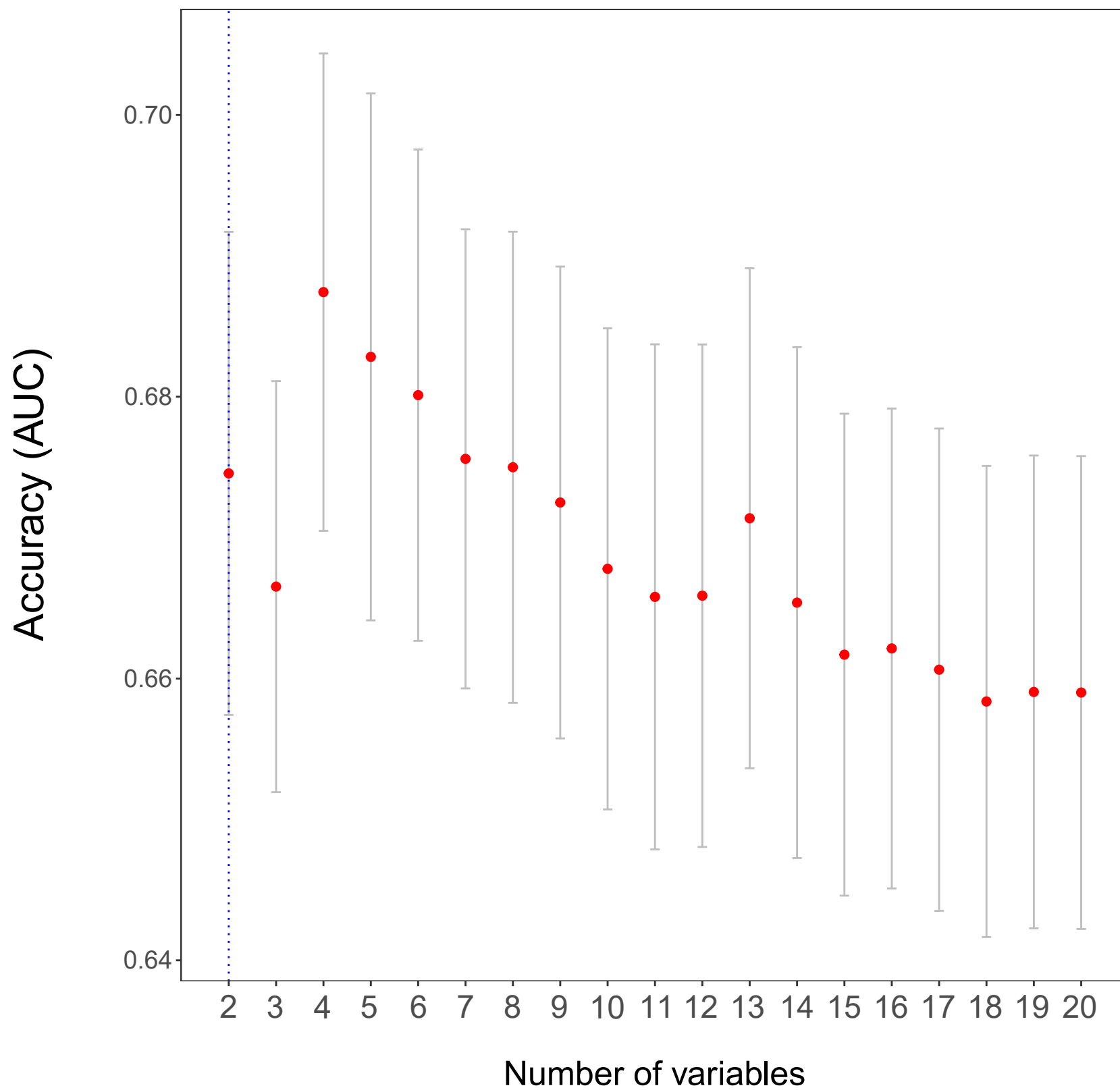

Supplement: FIG S3 [file sys004182245sf3.pdf]

# Figure S4

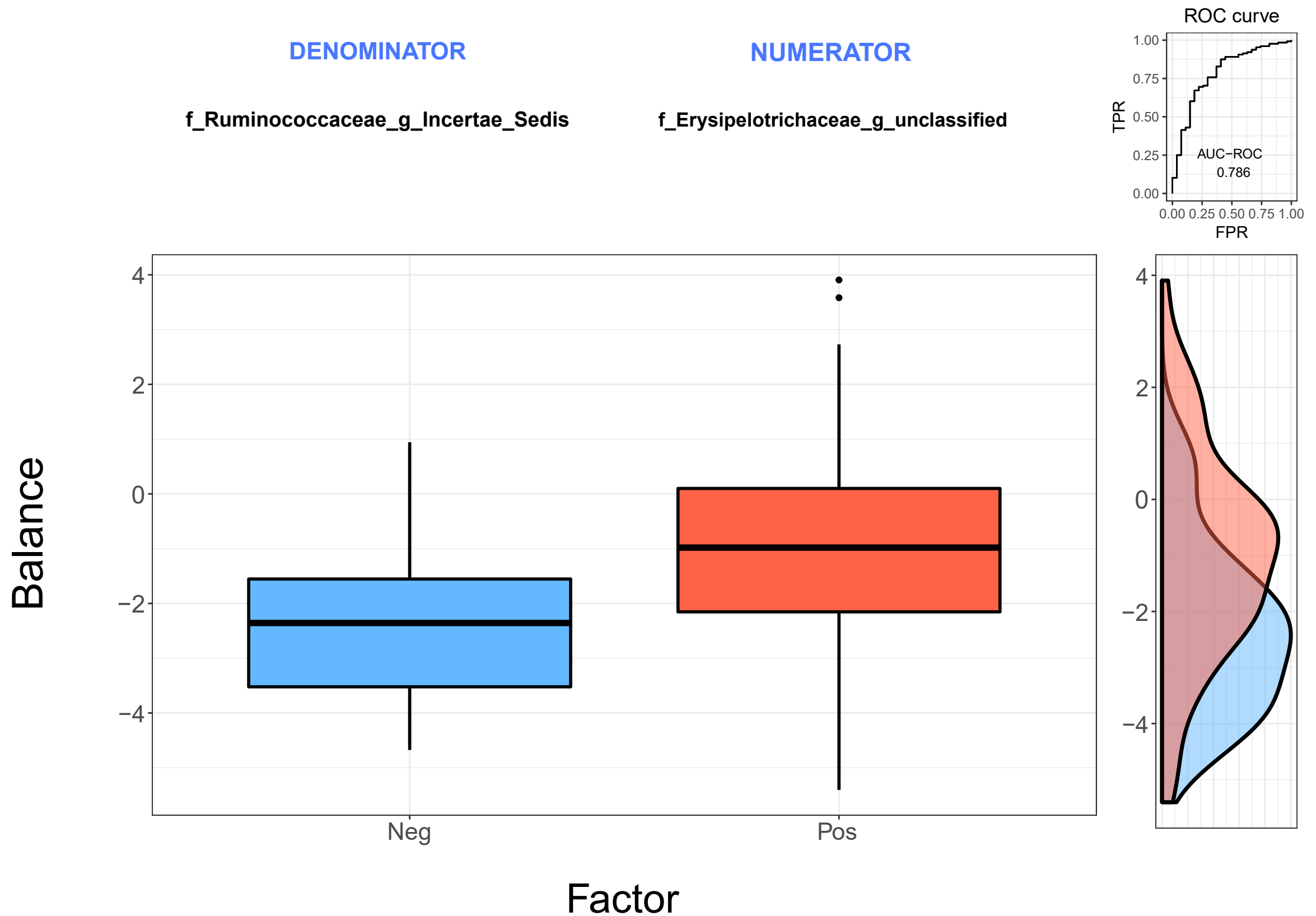

Supplement: FIG S4 [file sys004182245sf4.pdf]

# Figure S6

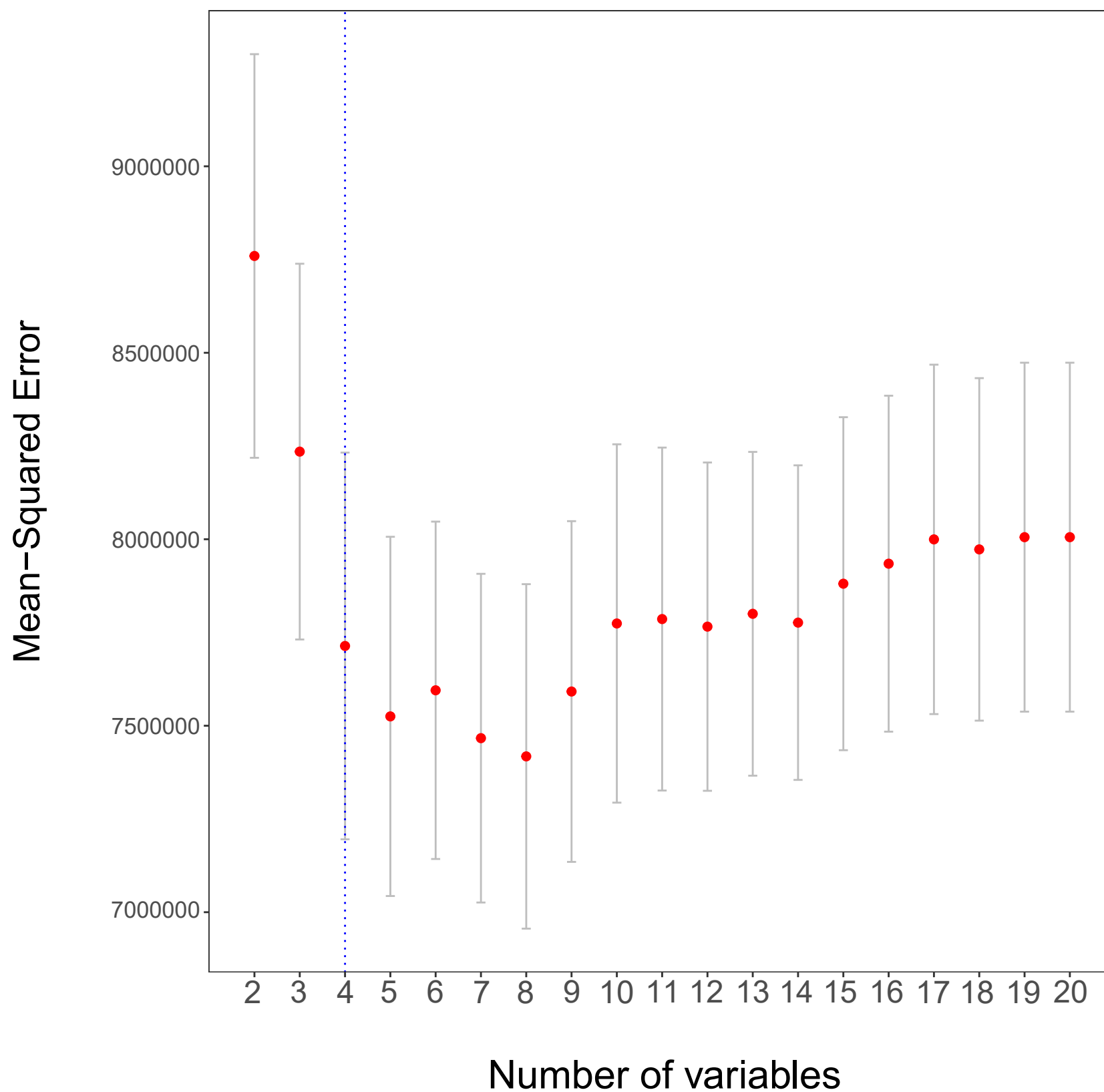

Supplement: FIG S6 [file sys004182245sf6.pdf]

# Figure S7

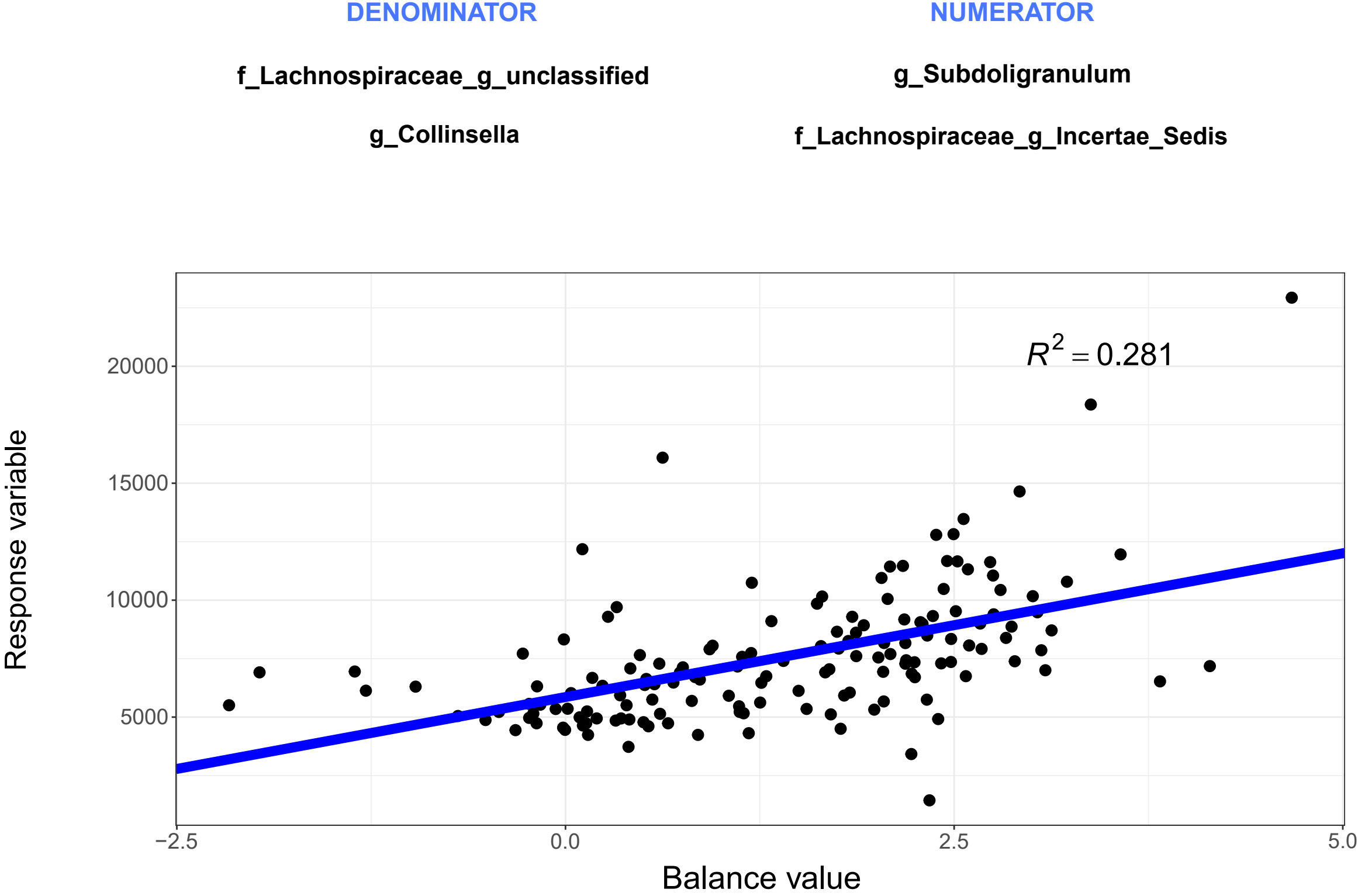

Supplement: FIG S7 [file sys004182245sf7.pdf]
