# Supplementary material for: Balances: a New Perspective for Microbiome Analysis
Source: mSystems. 2018 Jul 17;3(4):e00053-18. doi: 10.1128/mSystems.00053-18 (PMC6050633; doi:10.1128/mSystems.00053-18)
Supplement: FIG S5 [file sys004182245sf5.pdf]

Figure S5

	%	Global	BAL 1	BAL 2	BAL 3
f_Ruminococcaceae_g_Incertae_Sedis	76				
f_Erysipelotrichaceae_g_unclassified	50				
g_Bacteroides	30				
g_Phascolarctobacterium	12				
FREQ	–	–	0.44	0.24	0.06
