# Supplementary material for: Balances: a New Perspective for Microbiome Analysis
Source: mSystems. 2018 Jul 17;3(4):e00053-18. doi: 10.1128/mSystems.00053-18 (PMC6050633; doi:10.1128/mSystems.00053-18)
Supplement: FIG S8 [file sys004182245sf8.pdf]

Figure S8

	%	Global	BAL 1	BAL 2	BAL 3
f_Lachnospiraceae_g_unclassified	94				
g_Collinsella	76				
g_Subdoligranulum	72				
f_Lachnospiraceae_g_Incertae_Sedis	54				
g_Thalassospira	50				
g_Bifidobacterium	14				
FREQ	–	–	0.34	0.12	0.08
